# The Cinematograph in Medicine

**Published:** 1907-11-30

**Authors:** C. O. Hawthorne

**Affiliations:** Harley Street, W.


### EDITOR'S LETTER-BOX.

**Our Correspondents are reminded that prolixity is a great bar to publication, and that brevity of style and conciseness of statement greatly facilitate early insertion.]**

### THE CINEMATOGRAPH IN MEDICINE.

SIR,—In your issue of November 23 (p. 200) it is stated that the cinematograph was first pressed into the service of medicine "at the last Berlin Congress." I must confess that I am not certain of the date of this Congress, but I assume the reference to be to a comparatively recent event. Allow me, then, to point out that so long ago as 1897 a cinematograph demonstration of the characteristic peculiarities of the gait in various forms of organic nervous disease was given to the Glasgow Medico-Chirurgical Society by Dr. Donald J. Mackintosh, the medical superintendent of the Glasgow Western Infirmary. A note of this fact appears on page 287 in volume i. of the Society's Transactions.

Yours, etc.,

Harley Street, W. C. O. HAWTHORNE.

**THE HOSPITAL** *November 30, 1907.*

